# Predictors of success of pharmacological management in patients with chronic lower back pain: systematic review

**DOI:** 10.1186/s13018-024-04741-9

**Published:** 2024-04-18

**Authors:** Alice Baroncini, Nicola Maffulli, Michael Mian, Raju Vaishya, Francesco Simeone, Filippo Migliorini

**Affiliations:** 1 GSpine4, IRCCS Ospedale Galeazzi – Sant’Ambrogio, Milan, Italy; 2 Department of Trauma and Orthopaedic Surgery, Faculty of Medicine and Psychology, University La Sapienza, 00185 Rome, Italy; 3 Barts and the London School of Medicine and Dentistry, Centre for Sports and Exercise Medicine, Queen Mary University of London, Mile End Hospital, London, E1 4DG England; 4 School of Pharmacy and Bioengineering, Keele University Faculty of Medicine, Thornburrow Drive, Stoke on Trent, England; 5 Innovation Research Teaching Service (IRTS), Academic Hospital of Bolzano (SABES-ASDAA), Teaching Hospital of the Paracelsus Medical University, 39100 Bolzano, Italy; 6 Department of Orthopaedics and Joint Replacement Surgery, Indraprastha Apollo Hospital, Sarita Vihar, New Delhi, 110076 India; 7 Department of Orthopedics and Trauma Surgery, Academic Hospital of Bolzano (SABES-ASDAA), Teaching Hospital of the Paracelsus Medical University, 39100 Bolzano, Italy; 8 Department of Orthopaedic, Trauma and Reconstructive Surgery, RWTH Aachen University Hospital, Pauwelsstraße 30, 52074 Aachen, Germany

**Keywords:** Low back pain, Risk factors, Pain, Management

## Abstract

**Background:**

Conservative management is recommended as the first therapeutic step in chronic low back pain (LBP), but there is no available evidence regarding the possible effect of patients’ baseline characteristics on the therapeutic outcomes. A systematic review of the literature was performed to investigate this point.

**Methods:**

In February 2024, all the level I studies investigating the role of pharmacological management for chronic LBP were accessed. Data concerning the patient demographic at baseline were collected: number of patients and related mean BMI and age, duration of the symptoms, duration of the follow-up, percentage of females, Numeric Rating Scale (NRS), the Roland Morris Disability Questionnaire (RMQ), Oswestry Disability Index (ODI). The outcomes at the last follow-up were evaluated through NRS, RMQ, and ODI. A multiple linear model regression diagnostic through the Pearson Product-Moment Correlation Coefficient (*r*) was used.

**Results:**

Data from 47 articles (9007 patients) were obtained. The analysis yielded the following significant associations: age at baseline and NRS at follow-up (*r* = − 0.22; *P* = 0.04), NRS at baseline with NRS (*r* = 0.26; *P* = 0.03) and RMQ (*r* = − 0.58; *P* = 0.02) at follow-up, RMQ at baseline and the same at follow-up (*r* = 0.69; *P* = 0.0001).

**Conclusion:**

Older age, higher BMI, presence of comorbidities, higher ODI and a long history of symptoms or surgical treatments do not reduce the efficacy of pharmacological management of chronic LBP. However, pharmacological therapy is not an effective option for patients with high baseline RMQ.

**Level of evidence:**

I systematic review of RCTs.

## Introduction

Chronic low back pain (LBP) is one of the leading causes of disability worldwide [1, 2]. 40% of LBP is discogenic in nature [3], but in many patients a specific cause of pain cannot be identified [4]. Chronic LBP is increasingly prevalent and has an increasing socioeconomic impact. In the United States, over 80% of the population will experience one episode of chronic LBP during their lifetime [5]. While the majority of cases are self-limiting, 20–44% of the affected population will develop chronic symptoms [6]. The economic costs of LBP are estimated at between US$ 100–200 billion annually, mainly from loss in wages and productivity of the affected patients [7]. Conservative management of LBP, the first therapeutic step in international guidelines [8, 9], mainly consists of physical therapy and pharmacological management. Nonpharmacologic management is recommended as the first treatment option, but this can prove inadequate for some patients and, in this case, pharmacological therapy should be initiated [9]. Several therapeutic options are available and include non-steroidal anti-inflammatory drugs (NSAIDs), tricyclic antidepressants selective serotonin reuptake inhibitors (SSRIs), and opioids [9, 10]. A holistic approach to the management of patients with chronic LBP is required, and it includes the evaluation of social and psychological factors or expectations [8]. However, there is no available evidence regarding the possible effect of patients’ characteristics such as age, body mass index (BMI), gender, duration of symptoms and follow-up on the responsiveness to pharmacological therapy. The evaluation of a patient’s specific features and the identification of possible risk factors which may reduce the effectiveness of a given therapeutic approach is of paramount importance to tailor the management of the patient’s needs. Therefore, a systematic review of the literature was performed to investigate whether patients’ baseline characteristics influence the efficacy of pharmacological management in terms of pain and disability. In this way, it would be possible to highlight negative or positive prognostic factors to guide healthcare professionals in identifying the ideal candidate for pharmacological management of chronic LBP. A multiple linear model analysis was conducted to investigate the impact of duration of follow-up, age, gender, BMI, symptoms duration before treatment, previous surgery, comorbidities, pain and disability pre-treatment on pain and disability at the last available follow-up.

## Methods

### Eligibility criteria

All the randomised controlled trials (RCTs) investigating the role of pharmacological treatments for chronic LBP were accessed. Articles in English, German, Italian, French and Spanish, according to the authors language capabilities, were eligible. According to the Oxford Centre of Evidence-Based Medicine (OCEBM) [12], only level I of evidence were considered. Articles treating patients with diagnosed psychiatric disorders were included. Articles treating patients with any type or form of adjuvants were excluded. Studies reporting data on patients with neurological, mechanical or non-specific LBP were eligible. Studies reporting data on patients with acute LBP were excluded, along with those reporting data concerning the cervicothoracic or sacroiliac spine tracts. Reviews, letters, registers, case reports, editorials, and expert opinions were excluded. Animal, cadaveric and biomechanics studies were also excluded. Only articles reporting quantitative data on the patient-reported outcome measures (PROMs) at the last follow-up were eligible. Missing data under the outcomes of interest warranted the exclusion from this study.

### Search strategy

The present study was conducted according to the Preferred Reporting Items for Systematic Reviews and Meta-Analyses: the PRISMA statement [11]. The search strategy was the following:

- P (population): chronic low back pain;
- I (intervention): medical treatments;
- C (correlation): patient demographic, therapy protocol and clinical scores;
- O (outcomes): Pain and disability.

### Data source

In February 2024, the literature search was performed by two authors (A.B. & F.S.) independently. The following databases were accessed: PubMed, Google Scholar, Embase, and Scopus. The following keywords were used in combination: *low, lumbar, spine, back, pain, disability, therapy, treatments, drugs, medication, medicine, conservative, tricyclic antidepressants, acetaminophen, amoxicillin, flupirtine, baclofen, atc, bupropion, ssri, topiramate, gabapentinoids, selective serotonin reuptake inhibitors, opioids, gabapentin, pregabalin, celecoxib, etoricoxib, valdecoxib, naproxen, diclofenac, nsaid, coxib, selective, non-selective, visual analogic scale, numeric rating scale, roland morris questionnaire, oswestry disability index*. The same authors screened the resulting papers for inclusion. The article full-text was accessed for eligible articles. A cross reference of the bibliographies was even performed. The process of data source and extraction was supervised by a third senior author (N.M.).

### Data extraction

Two authors (A.B. & F.S.) independently performed data extraction. Study generalities (author, year, journal, type of study) and data concerning the patient demographic were collected: number of patients and related mean BMI and age, duration of the symptoms and of the follow-up (months), women (%), previous surgery (n), and comborbiditites (n). Data on the following PROMs were retrieved at baseline and at the last follow-up: Numeric Rating Scale (NRS), the Roland Morris Disability Questionnaire (RMQ) [13], Oswestry Disability Index (ODI) [14]. Data on the following PROMs were retrieved at last follow-up: NRS, RMQ, ODI.

### Methodology quality assessment

For the methodological quality assessment, the risk of bias summary graph of the Review Manager Software (The Nordic Cochrane Collaboration, Copenhagen) was performed by two authors (A.B. & F.S.) independently. The following risks of bias were evaluated for analysis: selection, detection, reporting, attrition, and other source of bias.

### Statistical analysis

The statistical analyses were performed by one author (F.M.). The baseline was assessed through the IBM SPSS software version 25. To assess whether factors at baseline (length of the follow-up symptoms duration, age, BMI, sex, previous surgery, comorbidities, PROMs) exert an influence on pain and disability at the last follow-up, a multiple linear model regression diagnostic was conducted. The STATA/MP 16.1 (StataCorp, College Station, TX) software was used. For pairwise correlation, the Pearson Product-Moment Correlation Coefficient (*r*) was used. The final effect ranked between + 1 (positive linear correlation) and − 1 (negative linear correlation), according to the Cauchy–Schwarz inequality. Values of 0.1 <| *r* |< 0.3, 0.3 <|*r*|< 0.5, and |*r*|> 0.5 were considered to have small, moderate, and strong correlations, respectively. The test of overall significance was performed through the χ^2^ test, with values of *P* < 0.05 considered statistically significant.

## Results

### Search result

**Fig. 1:**
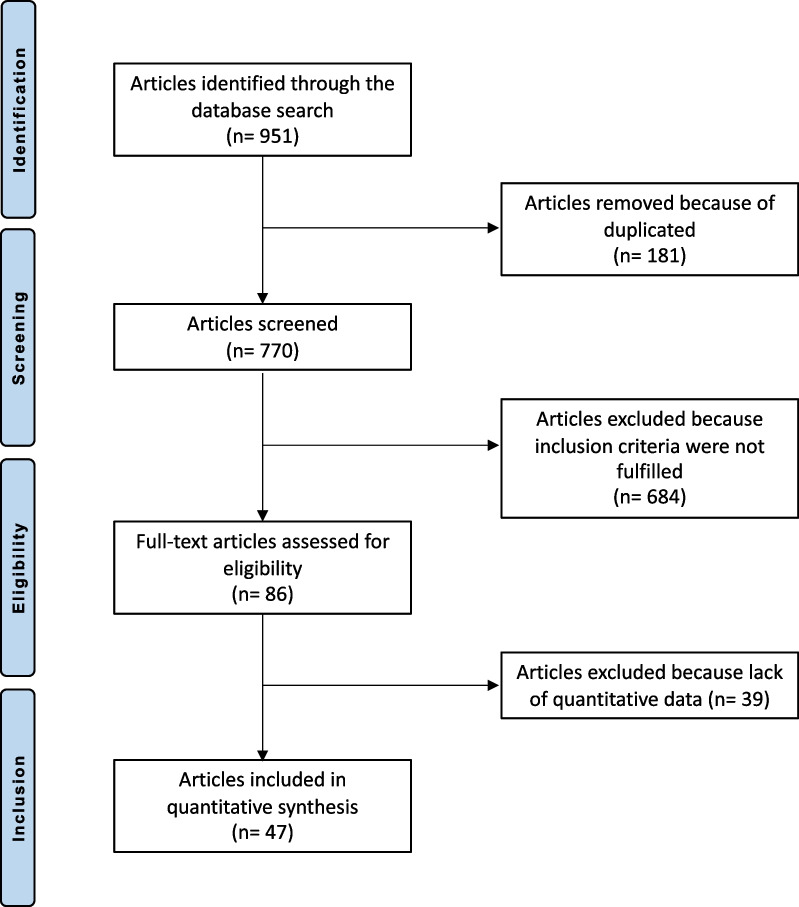
PRISMA flow chart of the literature search

The literature search resulted in 2701 articles. Of them, 951 were RCTs, with 181 duplicates. A further 684 articles were not eligible: surgical waiting list articles (N = 2), not matching the topic (N = 231), major trauma, deformities, neurologic disorders, comorbidities or uncontrolled medical illness (N = 138), combined treatments (N = 112), spondylodiscitis or other infective illness (N = 37), language limitation (N = 45), acute onset LBP (N = 49), cervicothoracic or sacroiliac pain (N = 41), other (N = 29). Given the lack of data on the outcomes of interests, a further 39 RCTs were excluded. This left 47 articles for review. The literature search results are shown in Fig. 1.

### Methodological quality assessment

**Fig. 2:**
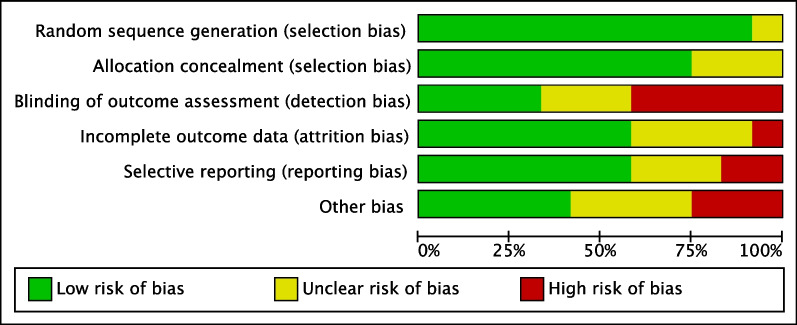
Cochrane risk of bias graph

The risk of selection bias was low, reflecting the selective inclusion of RCTs. Two-thirds of the included papers performed sample blinding, thus leading to a poor to moderate risk of detection bias. The overall good quality of the included studies results in a low risk of reporting, attrition and other biases. Concluding, the quality of the methodological assessment scored good. The Cochrane graph of the bias tool is shown in Fig. 2.

### Patient demographics

Data from 9007 patients (mean age 52.6 ± 7.0 years; mean BMI: 28.3 ± 2.8 kg/m^2^; mean duration of symptoms before beginning treatment: 81.2 ± 46.2 months; mean follow-up: 3.2 ± 3.2 months) were obtained. At baseline, NRS scored 55.3 ± 19.5, RMQ 10.67 ± 2.6, and ODI 37.20 ± 11.1. Patient demographics are shown in Table 1.

**Table 1:** Generalities and demographic baseline of the included studies

Author, year	Journal	Follow-up (months)	Treatment	Patients (n)	Mean age	Women (%)	Symptoms duration (months)
Allan et al. [15]	*Spine*	13.0	Fentanyl	338	53.4	61.0	122.5
			Morphine	342	54.7	38.0	127.0
Atkinson et al. [16]	*Pain*	2.0	Maprotiline	33	49.2	36.9	174.0
			Paroxetine	34			
			Placebo	36			
Atkinson et al. [17]	*Pain*	3.0	Gabapentin	55	57.8	18.9	206.0
			Placebo	53	54.6	24.5	213.5
Baron et al. [18]	*Pain Practice*	2.8	Tapentadol	154	58.5	61.5	112.8
			Tapentadol & Pregabalin	159	56.3	54.1	104.4
Bedaiwi et al. [4]	*Arthritis Care & Research*	1.0	Acetaminophen	25	37.2	44.0	
			Celecoxib	25	43.4	52.0	
Birbara et al. [19]	*J Pain*	3.0	Etoricoxib	101	52.3	63.4	145.2
			Etoricoxib	106	52.2	63.2	129.6
			Placebo	107	51.0	55.1	128.4
Bråten et al. [20]	*BMJ*	12.0	Amoxicillin	89	44.7	60.0	36.0
			Placebo	91	45.2	57.0	40.8
Buynak et al. [21]	*Expert Opin. Pharmacother*		Oxycodone	328	50.0	55.2	
			Tapentadol	318	49.4	61.0	
			Placebo	319	50.4	57.7	
Chu et al. [22]	*Pain*	1.0	Morphine	69	44.0	36.2	
			Placebo	70	46.0	51.4	
Coats et al. [23]	*Clinical Therapeutics*	1.0	Valdecoxib	148	48.6	54.7	139.2
			Placebo	145	48.7	58.6	130.8
Gordon et al. [24]	*Clin Ther*	2.0	Buprenorphine	39	50.7	60.3	154.8
			Placebo	39			
Hale et al. [25]	*J Pain*	3.0	Oxymorphone Release	70	48.2	57.1	
			Placebo	72	46.0	33.3	
Hwang et al. [26]	*Pain Res and Man*	1.9	Fentanyl	52	59.0	57.7	
			Gabapentin	56	58.2	53.6	
Jamison et al. [27]	*SPINE*	4.0	Naproxen	12	42.6	57.1	79.1
			Oxycodone	13			
			Oxycodone & Morphine	11			
Kalita et al. [28]	*J Neurological Sci*	3.5	Amitriptyline	103	41.6	45.5	35.2
			Pregabalin	97	42.0		35.9
Katz et al. [29]	*Am J Pain*	4.0	Bupropion	21	49.8	57.1	
			Placebo	23	51.4	39.1	
Katz et al. [30]	*Curr Med Res Opin*	3.0	Oxymorphone	105	51.3	56.2	
			Placebo	100	48.1	50.0	
Khoromi et al. [31]	*J Pain*	4.5	Topiramate & Placebo	21			
			Placebo & Topiramate	20			
Klinger et al. [32]	*PAIN*		Opioid	12	50.0	18.75	119.33
			Opioid & Conditioning	12	53.0	18.75	158.83
			Placebo	12	50.8	18.75	165.67
			Placebo & Conditioning	12	50.3	18.75	162.17
Konno et al. [33]	*SPINE*	3.5	Placebo	226	57.8	54.0	123.6
			Duloxetine	230	60	50.0	117.6
Krebs et al. [34]	*JAMA*	12.0	Opioid	120	56.8	13.0	
			Non-Opioid group	120	59.7	13.0	
Markman et al. [35]	*Neurology*	1.0	Pregabalin & diphenhydramine	14	71.1	29.0	
			Diphenhydramine & pregabalin	15	69.0	33.0	
Muehlbacher et al. [36]	*Clin J Pain*	2.5	Topiramate	48	48.8	39.6	
			Placebo	48	48.7	35.4	
Perrot et al. [37]	*Clin Ther*	0.3	Paracetamol & Tramadol	59	56.5	64.4	
			Tramadol	60	54.1	51.7	
Pheasant et al. [38]	*SPINE*		Atropine	6	47.2	75.0	118.8
			Amitriptyline	10			
Pota et al. [39]	*Pain Manage*	1.5	Buprenorphine & pregabalin	22	55.0	50.0	15.0
			Buprenorphine	22			
Robertson et al. [40]	*JAMA Neur*	2.0	Pregabalin	10	57.0	39.0	
			Gabapentin	8			
Romano et al. [41]	*J Orthop Traumatol*	3.0	Several protocols combining Pregabalin, Celecoxib and Placebo	36	53.0	55.6	
Ruoff et al. [42]	*Clinical Therapeutics*	3.0	Tramadol & Acetaminophen	161	53.6	67.1	
			Placebo	157	54.1	59.2	
Sakai et al. [43]	*Eur Spine J*		Pregabalin	30	72.0	30.0	
			Tramadol & Acetaminophen	30	72.6	36.7	
Schiphorst Preuper et al. [44]	*Eur Spine J*	0.5	Tramadol & Acetaminophen	25	42.0	72.0	18.0
			Placebo	25	44.0	64.0	24.0
Schliessbach et al. [45]	*European J Pain*		Clobazam	49	54.3	59.0	145.2
			Placebo				
Schliessbach et al. [46]	*PLoS ONE*		Imipramine	50	54.4	64.0	134.4
			Placebo				
Schliessbach et al. [47]	*European J Pain*		Oxycodone	50.0			
			Imipramine	50.0			
			Clobazam	49.0			
Schukro et al. [48]	*Anesthesiology*	1.0	Duloxetine	25	57.9	51.0	18.0
			Placebo				
Shell et al. [49]	*Am J Ther*	1.0	Naproxen	43			
			Theramine	42			
			Naproxene & Theramine	44			
Skljarevski et al. [50]	*J Pain*	3.0	Placebo	203	53.4	63.1	104.4
			Duloxetine	198	54.9	59.6	99.6
Skljarevski et al. [51]	*Pain Medicine*	9.0	Duloxetine	83	51.2	65.1	104.4
			Duloxetine & Placebo	98	52.2	63.3	120.0
Steiner et al. [52]	*J Pain Symptom Management*	3.0	Buprenorphine	257	48.8	52.0	
			Placebo	284	50.0	58.0	
Takahashi et al. [53]	*Fukushima J Med Sci*	3.0	NSAID	15	53.3	53.3	5.92
			Control group	18	57.6	55.6	7.63
Tetsunaga et al. [54]	*J Orthop Sci*	2.0	Tramadol	35	65.4	62.9	46.0
			NSAID	35	62.3	62.9	54.2
Überall et al. [55]	*Curr Med Res Opin*	1.0	Placebo	120	59.2	55.8	76.4
			Flupirtine	119	58.6	68.9	69.2
			Tramadol	116	57.6	61.2	71.7
Urquhart et al. [56]	*JAMA Intern Med*	6.0	Amitriptyline	72	53.5	39.0	159.6
			Control group	74	56.0	38.0	182.4
Webster et al. [57]	*J Pain*	3.0	Placebo	101	48.7	61.4	
			Oxycodone	206	47.9	61.2	
			Oxytrex	206	47.8	61.7	
			Oxytrex	206	47.9	61.7	
Yang et al. [58]	*Yonsei Med J*	0.5	Aceclofenac	50	57.6	76.0	50.0
			Aceclofenac	50	56.9	76.0	91.6
Zaringhalam et al. [59]	*Chinese Med*	2.5	Baclofen	21	55.1		80.4
			Acupuncture	21	54.2		85.2
			Baclofen & Acupuncture	21	54.2		82.8
			Control group	21	54.3		84.0
Zerbini et al. [60]	*Curr Med Res Opin*	1.0	Etoricoxib	224	51.7	71.9	99.6
			Diclofenac	222	52.2	71.6	99.6

### Outcomes of interest

No evidence of a statistically significant association was observed between NRS, RMQ and ODI at the last follow-up and other parameters such as symptom duration before treatment, duration of the follow-up, gender, BMI, comorbidities and previous surgeries. Patients’ age at baseline showed evidence of a statistically significant negative association with the NRS (*r* = − 0.22; *P* = 0.04). The ODI at baseline also did not influence any of the outcomes at the last follow-up (*P* > 0.1). The NRS at baseline was associated proportionally with NRS (*r* = 0.26; *P* = 0.03) and RMQ (*r* = − 0.58; *P* = 0.02) at follow-up. The RMQ at baseline showed evidence of a statistically significant association with the RMQ at the last follow-up (*r* = 0.69; *P* = 0.0001). An overview of the main results is shown in Table 2.

**Table 2:** Overview of the main results of the multivariate analysis

Endpoint	Observations	Numeric rating scale		Roland–Morris questionnaire		Oswestry disability index	
		*r*	P	*r*	P	*r*	P
Follow-up (months)	4910	0.22	0.1	0.01	0.9	− 0.25	0.3
Age	8830	− 0.22	0.04	− 0.01	0.9	− 0.14	0.6
Female gender	5125	0.09	0.5	− 0.26	0.3	− 0.45	0.09
BMI	2701	0.01	0.9	− 0.07	0.8	0.86	0.1
Symptoms duration (months)	3452	0.08	0.6	− 0.32	0.2	− 0.26	0.7
Previous surgery	180	1.00	1.00	1.00	1.00	1.00	1.00
Comorbidities	625	0.01	0.9				
NRS baseline	3943	0.26	0.03	− 0.58	0.02	0.12	0.7
RMQ baseline	2657	0.21	0.4	0.69	0.0001	0.60	0.4
ODI baseline	1109	0.20	0.5	0.25	0.8	0.17	0.6

## Discussion

The main results of the present study were that in patients with chronic LBP, higher pain and disability levels at baseline showed evidence of a statistically significant association with poorer outcomes at the last follow-up, while a negative, albeit weak, association was observed between age and NRS. Other factors such as gender, BMI, comorbidities, duration of symptoms and previous surgeries did not have any influence on the effectiveness of the pharmacological therapy.

To our knowledge, this is the first study to assess the effects of patient baseline characteristics on the outcomes of the pharmacological management of chronic LBP. Given the availability of different treatment options for this condition, identifying the factors that, positively or negatively, are associated with the outcomes of a given therapy is paramount to offer patients the management with the highest likelihood of success based on their specific characteristics.

One previously published work highlighted numerous demographic, social and psychological factors associated with LBP [61]. Among these, older age and the consequent decline in mobility and cognitive function were reported to be possible risk factors for developing LBP [10]. Such features may suggest a worse prognosis for older patients, but this hypothesis was surprisingly not confirmed by the results of the present investigation. Even though the negative association between age and NRS was weak, and thus it may not be inferred that older patients show better outcomes than younger ones, it is safe to conclude that older age does not exert a negative impact on the effects of pharmacological management of LBP. Nonetheless, when planning pharmacological therapy for older individuals, it is of paramount importance to consider possible comorbidities and impairment of the renal and hepatic function to prevent potentially serious adverse events [10, 61].

Chronic LBP is reported to be more prevalent among women [1, 62, 63]. However, the available data show no association between gender and pain and disability levels after pharmacological therapy. While this management had the same effects on the male and female population, it is important to bear in mind the differences in the biomechanical and aetiologic backgrounds of LPB between the genders [64, 65], as these may be specifically targeted with additional physiotherapy or psychological support to tailor the therapy on the patients’ needs. Further research is required to investigate this topic.

One clinically relevant finding of the current study was that the duration of symptoms and previous spinal surgery did not exert a negative effect on the outcomes at the last follow-up after pharmacological therapy. This observation is relevant both for the treating physician, as a long course of unsatisfactory symptom control does not translate into a poorer prognosis, and for the patients. Pain-related fear and the perception of treatment failure contribute to a vicious cycle which potentially increases pain perception and disability [66, 67]. While specific psychological therapies can and should be employed to target this particular issue, an optimistic view, giving the message that previous treatment failure does not impact on the future outcome and prognosis, may represent a first step in reassuring the patients with a long history of spinal symptoms.

Notably, the RMQ at the last follow-up showed a strong association with the NRS and RMQ at baseline. This finding suggests that pharmacological management may not be sufficient to target the limitations that patients with chronic LBP encounter in their daily activities. Thus, in patients with consistent disability at baseline, a multimodal approach may be more successful [68]. Another interpretation of this finding is that the ODI offers a more nuanced scale (how much does an item apply to the patient) rather than a yes/no answer such as in the RMQ. Thus, after pharmacological management, a patient could be “less impaired” in the activities of daily living but still experience some limitations. While this can be highlighted with the use of the ODI, the results of the RMQ would likely be similar before and after treatment. Unfortunately, the literature regarding this aspect is still lacking, and the evidence concerning the most effective physiotherapeutic interventions is still low [69]. Further studies are required to investigate this point.

Many compounds are available for the pharmacological management of chronic LBP, including opioids, NSAIDs, and tricyclic antidepressants. Peck et al. [70] have published a comprehensive review of over-the-counter medication for the treatment of low back pain: all may be effective, but it is unclear whether one is more effective than the others. A recent systematic review observed that NSAIDs, duloxetine, baclofen and opiates are the most effective drugs in the setting of chronic LBP [10]. This highlights an area of future research, as different prognostic factors may be analysed concerning every specific treatment option. However, the available literature does not currently present sufficient data for this analysis, and thus represents a limitation of this work.

Another limitation of this study was that patients with neurological, mechanical and non-specific LBP were included. While a different aetiology may lead to a different response to the pharmacological management and thus to a different weight of prognostic factors on pain and disability, the available data did not allow a separate analysis for the different causes of LBP as the included investigations did not present separate outcomes for the different aetiologies included. Similarly, patients with concomitant psychological disorders were also included in the analysis: while this might represent a possible confounding factor, the authors opted to maintain these patients in the work to allow for sufficient numerosity. However, it is important to highlight that LBP is often managed by general practitioners and, upon successful initial treatment, a further investigation of the cause of LBP is not performed. Thus, despite possibly including a source of bias, the heterogeneity of the data reflects the clinical practice. Lastly, the available data were insufficient to perform a sub-analysis of possible differences among the predictive factors in the various considered pharmaceutic compounds. During the database search and data extraction, the inter-agreement rate was not evaluated.

## Conclusion

Pharmacological management of LBP can yield good results also in older patients, in those with a higher BMI or other comorbidities, or patients with a long history of symptoms or previous surgical treatment. There are no differences between the genders in the effectiveness of pharmacological management. Patients who present with a high RMQ score are likely to maintain high RMQ values after treatment. A precise definition of the baseline traits influencing therapeutic outcomes in patients undergoing pharmacological therapy for chronic LBP is an essential step to tailor management to the specific needs and characteristics of the patient.

## Data Availability

The data underlying this article are available in the article and in its online supplementary material.

## Abbreviations

NRS: Numeric Rating Scale
RMQ: The Roland Morris Disability Questionnaire
ODI: Oswestry Disability Index
PROMs: Patient-reported outcome measures
RCT: Randomised clinical trial
BMI: Body mass index
NSAID: Nonsteroidal anti-inflammatory drugs
LBP: Low back pain
